# The Use of a Non-Conventional Long-Lived Gallium Radioisotope ^66^Ga Improves Imaging Contrast of EGFR Expression in Malignant Tumours Using DFO-ZEGFR:2377 Affibody Molecule

**DOI:** 10.3390/pharmaceutics13020292

**Published:** 2021-02-23

**Authors:** Maryam Oroujeni, Tianqi Xu, Katherine Gagnon, Sara S. Rinne, Jan Weis, Javad Garousi, Ken G. Andersson, John Löfblom, Anna Orlova, Vladimir Tolmachev

**Affiliations:** 1 Department of Immunology, Genetics and Pathology, Uppsala University, 75185 Uppsala, Sweden; maryam.oroujeni@igp.uu.se (M.O.); tianqi.xu@igp.uu.se (T.X.); javad.garousi@igp.uu.se (J.G.); 2 GE Healthcare, GEMS PET Systems, 75015 Uppsala, Sweden; katherine.gagnon@ge.com; 3 Department of Medicinal Chemistry, Uppsala University, 75183 Uppsala, Sweden; sara.rinne@ilk.uu.se (S.S.R.); anna.orlova@ilk.uu.se (A.O.); 4 Department of Medical Physics, Uppsala University Hospital, 75185 Uppsala, Sweden; jan.weis@radiol.uu.se; 5 Department of Protein Science, KTH Royal Institute of Technology, 10691 Stockholm, Sweden; ken2@kth.se (K.G.A.); lofblom@kth.se (J.L.); 6 Research Centrum for Oncotheranostics, Research School of Chemistry and Applied Biomedical Sciences, Tomsk Polytechnic University, 634050 Tomsk, Russia

**Keywords:** epidermal growth factor receptor, affibody molecule, PET imaging, gallium-66, ZEGFR:2377, A431 xenograft

## Abstract

Epidermal growth factor receptor (EGFR) is overexpressed in many malignancies. EGFR-targeted therapy extends survival of patients with disseminated cancers. Radionuclide molecular imaging of EGFR expression would make EGFR-directed treatment more personalized and therefore more efficient. A previous study demonstrated that affibody molecule [^68^Ga]Ga-DFO-ZEGFR:2377 permits specific positron-emission tomography (PET) imaging of EGFR expression in xenografts at 3 h after injection. We anticipated that imaging at 24 h after injection would provide higher contrast, but this is prevented by the short half-life of ^68^Ga (67.6 min). Here, we therefore tested the hypothesis that the use of the non-conventional long-lived positron emitter ^66^Ga (T_1/2_ = 9.49 h, β^+^ = 56.5%) would permit imaging with higher contrast. ^66^Ga was produced by the ^66^Zn(p,n)^66^Ga nuclear reaction and DFO-ZEGFR:2377 was efficiently labelled with ^66^Ga with preserved binding specificity in vitro and in vivo. At 24 h after injection, [^66^Ga]Ga-DFO-ZEGFR:2377 provided 3.9-fold higher tumor-to-blood ratio and 2.3-fold higher tumor-to-liver ratio than [^68^Ga]Ga-DFO-ZEGFR:2377 at 3 h after injection. At the same time point, [^66^Ga]Ga-DFO-ZEGFR:2377 provided 1.8-fold higher tumor-to-blood ratio, 3-fold higher tumor-to-liver ratio, 1.9-fold higher tumor-to-muscle ratio and 2.3-fold higher tumor-to-bone ratio than [^89^Zr]Zr-DFO-ZEGFR:2377. Biodistribution data were confirmed by whole body PET combined with magnetic resonance imaging (PET/MRI). The use of the positron emitter ^66^Ga for labelling of DFO-ZEGFR:2377 permits PET imaging of EGFR expression at 24 h after injection and improves imaging contrast.

## 1. Introduction

Several families of cell-surface receptors are overexpressed in malignant tumors. Signaling of the overexpressed receptors is frequently a driving force for malignancy. The epidermal growth factor receptor (EGFR) is one of four members of the human EGF receptor family of receptor tyrosine kinases [1]. EGFR activation normally causes signaling leading to cell proliferation, inhibition of apoptosis, and increased motility [2]. EGFR is overexpressed in several tumor types, including head and neck, breast, renal, non-small cell lung, colorectal, ovarian, pancreatic, and bladder cancers, thus triggering an early interest in EGFR-targeting therapies [3]. Today, EGFR is a well-established target for monoclonal antibodies such as cetuximab, panitumumab, and tyrosine kinase inhibitors such as gefitinib [1]. Overexpression of EGFR is associated with resistance against chemotherapy and radiotherapy and is also associated with poor prognosis in many cancers. Thus, the overexpression of EGFR could be a prognostic and predictive biomarker. It is critical to evaluate EGFR expression levels in tumors before treatment in order to select candidate patients for EGFR-targeting therapies.

Immunohistochemistry evaluation of a biopsy is the most common method to assess the expression level of EGFR. However, biopsying is an invasive method, which complicates sampling of multiple metastases. In addition, a high level of expression heterogeneity, discordance of EGFR expression level in primary tumors and metastases, and changes of EGFR expression during therapy make biopsy sampling unreliable [4,5,6]. Thus, it is necessary to develop an accurate and non-invasive method for detecting EGFR expression in order to select patients potentially responding to EGFR-targeted treatment.

Radionuclide molecular imaging, such as single photon emission computed tomography (SPECT) or positron-emission tomography (PET), is a potentially suitable method to detect EGFR expression level and circumvent challenges associated with biopsies. It has to be mentioned that radionuclide imaging of EGFR could be complicated due to the expression of this receptor by normal hepatocytes. Interaction with hepatocytes results in a rapid elimination of an imaging probe from blood and its insufficient bioavailability for a sensitive imaging [7]. However, Divgi and co-workers [7] have demonstrated in a clinical trial that co-injection of non-labelled antibody permits saturation of EGFR on hepatocytes thereby enabling good visualization of EGFR-expressing tumors by the ^111^In-labelled monoclonal antibody 225.

A pre-requisite for successful use of molecular imaging is the development of a suitable probe that provides sufficiently high sensitivity and specificity. Anti-EGFR monoclonal antibodies labelled with several nuclides [7,8,9,10,11,12,13] have been evaluated for EGFR visualization. Imaging sensitivity using monoclonal antibodies is, however, limited by their long blood residence and slow tumor accumulation thereby requiring several days (4–7 days) to obtain an acceptable imaging contrast in clinics [14,15]. Additionally, tumor uptake of unspecific antibodies might be up to 50% of specific ones [16,17], and has therefore been associated with a risk of false-positive diagnoses [18].

Reducing the size of the targeting proteins is a commonly used strategy to improve contrast and specificity of imaging [19]. One such example includes the use of imaging probes based on small engineered scaffold proteins such as affibody molecules [20,21]. Affibody molecules are small and robust three-helix proteins based on a 58-amino-acid scaffold derived from one of the immunoglobulin-binding domains of protein A. Affibody molecules with new specificities are generated by directed evolution, i.e., construction of combinatorial libraries followed by selections by, for example, phage display technology [20]. Affibody molecules are much smaller (molecular weight of 6–7 kDa) than monoclonal antibodies (150 kDa). Due to their small size, they show efficient extravasation and rapid tumor penetration. Importantly, unbound targeting agents are rapidly cleared from blood via the kidneys resulting in acquisition of high-contrast images after only a few hours post-injection [21,22]. Several affibody-based probes have been labelled previously with different radionuclides [23,24,25,26,27,28,29,30,31,32] for imaging of EGFR-expressing tumors.

The affibody molecule ZEGFR:2377 has previously been selected to have equal subnanomolar affinity to both human (0.9 nM) and murine (0.8 nM) EGFR [23]. This is an important aspect for assessment of biodistribution in mouse models with human xenografts [23]. We have found that injection of 30–50 µg of ^111^In-labelled DOTA-ZEGFR:2377 permits saturation of EGFR expression in the liver without blocking in tumor xenografts [23]. An important feature of the ZEGFR:2377 affibody molecule is its slow internalization after binding to the EGFR [23,27,31]. Thus, the majority of the specifically bound tracer remains on the cell surface of both tumor cells and hepatocytes in the liver and the rapid renal clearance of the probe shifts the binding equilibrium to dissociation. As a result, the liver, which is acting as a depot for ZEGFR affibody molecules, releases the labelled probe into the blood stream, in turn causing slower blood clearance compared with e.g., affibody molecules binding to HER2. As the tumor-to-blood ratio is significantly higher at 24 compared with 4 h after injection [23], research efforts aimed at developing an optimal label for ZEGFR:2377 affibody molecules have shifted to more long-lived radionuclides, such as ^55/57^Co (T_1/2_ = 17.5 h) [27], ^64^Cu (T_1/2_ = 12.7 h) [29,30], and ^89^Zr (T_1/2_ = 78.4 h) [31,32] as these could provide higher contrast for next-day PET imaging. These studies have provided important results including the finding that the combination of a 1,4,7,10-tetraazacyclododecane-1,4,7,10-tetraacetic acid (DOTA) chelator with either ^68^Ga or ^57^Co has been found to have a dramatic influence on hepatic uptake of ZEGFR:2377. This indicated that there is an EGFR-independent mechanism of the hepatic uptake that can be influenced by the charge of the chelator-metal complex [27].

**Figure 1:**
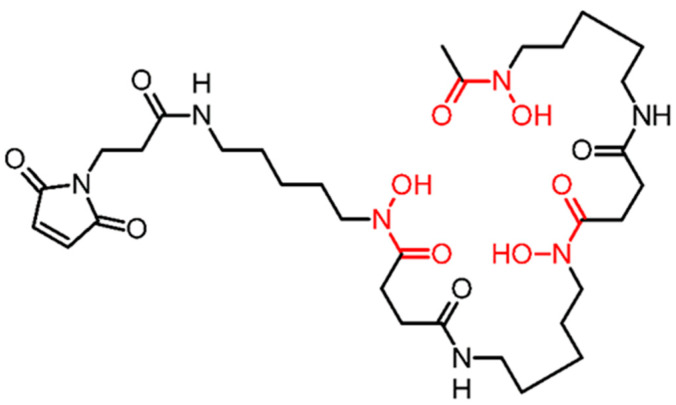
Structure of maleimide-derivatized desferrioxamine B (DFO).

Comparison of siderophore-based chelators fusarinine C (FSC) and desferrioxamine B (DFO) for labelling of ZEGFR:2377 affibody molecules with ^89^Zr also revealed an obvious influence of radionuclide–chelator combinations on biodistribution properties [32]; particularly, the use of DFO for high-temperature labelling provided better contrast. For site-specific labelling of ZEGFR:2377, a maleimido derivative of desferrioxamine B (*N*-[5-[[4-[5-[acetyl(hydroxy)amino]-pentylamino]-4-oxobutanoyl]-hydroxyamino]pentyl]-*N*0-(5-aminopentyl)-*N*0-hydroxybutanediamide, DFO) was conjugated to a unique C-terminal cysteine of this affibody molecule (Figure 1).

Several studies have shown that DFO is also a suitable chelator for radiolabeling of targeting agents with ^67^Ga or ^68^Ga [33,34,35]. DFO contains three hydroxamate groups, which provide six oxygen donors for the metal binding [36]. This is a good match for Ga^3+^, which is typically hexacoordinated. We have recently shown that ZEGFR:2377 affibody molecules labelled with ^68^Ga via DFO as a chelator have a tendency to demonstrate lower liver uptake and significantly increased tumor uptake when compared with ZEGFR:2377-DFO labelled with ^89^Zr at 3 h after injection [28]. This resulted in a higher tumor-to-liver ratio for the ^68^Ga-labelled conjugate. The use of Ga^3+^ instead of Zr^4+^ decreases the positive charge on C-terminus of ZEGFR:2377, which is favorable for biodistribution.

The use of long-lived radionuclides for radiolabeling of biomolecules undergoing slow distribution in vivo enables high-resolution imaging at later time points after injection. Gallium-66 (^66^Ga, T_1/2_ =9.49 h, β^+^ = 56.5%, EC = 43.5%) is a long-lived positron-emitting isotope of gallium [37]. The half-life of ^66^Ga makes it possible to perform next-day PET imaging, further improving imaging contrast. ^66^Ga has been tested for studies of some slow dynamic processes (such as lymphatic transport) using PET [38] and for imaging of tumor angiogenesis using a monoclonal antibody [39]. The use of a ^66^Ga-labelled somatostatin analogue as an imaging agent for somatostatin receptor positive tumors has also been reported [40]. Recently, first preclinical imaging of GRPR-expression in prostate cancer using [^66^Ga]Ga-NOTA-PEG2-RM26 has been reported [41].

The goal of this study was to test the hypothesis that the use of ^66^Ga would permit specific imaging of EGFR-expressing xenografts 24 h after injection and enhance the tumor-to-blood ratio when compared with imaging using the short-lived ^68^Ga radioisotope. For this purpose, labelling of DFO-ZEGFR:2377 affibody molecules with the positron emitting ^66^Ga radionuclide was optimized. The stability and in vitro targeting properties of [^66^Ga]Ga-DFO-ZEGFR:2377 were investigated and compared to targeting properties of previously studied ^68^Ga and ^89^Zr counterparts. In vivo targeting properties of a novel [^66^Ga]Ga-DFO-ZEGFR:2377 were compared directly with the properties of [^68^Ga]Ga-DFO-ZEGFR:2377 and [^89^Zr]Zr-DFO-ZEGFR:2377 at 3 and 24 h after injection, respectively. Such direct comparison is preferable to a comparison with historical controls as the targeting properties of all conjugates are therefore subject to the same animal batch-dependent biological parameters.

## 2. Materials and Methods

High quality Milli-Q water was used to prepare all buffer solutions, and the buffers were purified from metal contamination using Chelex 100 resin (Bio-Rad Laboratories, Hercules, CA, USA).

Gallium-68 (^68^Ga) was obtained by fractionated elution of the ^68^Ge/^68^Ga generator (Eckert and Ziegler AG, Berlin, Germany) with 0.1 M HCl. The eluate with the highest radioactivity concentration was used for labelling. Zirconium-89 (^89^Zr, solution in 1 M oxalic acid) was purchased from PerkinElmer (Waltham, MA, USA).

The radioactivity was measured using a gamma spectrometer with a NaI(Tl) detector (2480 WIZARD, Wallac Oy, Turku, Finland). The labelling yield and radiochemical purity of labelled conjugates were measured using the instant thin-layer chromatography (ITLC) (ITLC-SG, Agilent Technologies, Santa Clara, CA, USA). The strips were developed with 0.2 M citric acid, pH 2.0 and 5.0 for ^68^Ga and ^89^Zr conjugates, respectively. Distribution of the radioactivity among the strips was measured with a Cyclone Phosphor Storage Screen using OptiQuant software (Packard Instrument Company, Meriden, CT, USA) for data processing as well as Fujifilm Bioimaging Analyzers (BAS) 1800II using MultiGauge V3.0 analysis software (Tokyo, Japan). Radiolabelled bioconjugates were purified for further studies using NAP-5 size exclusion columns (Cytiva, Uppsala, Sweden) pre-equilibrated with phosphate-buffered saline (PBS) with 1% bovine serum albumin. In Vitro cell studies were performed using the EGFR-expressing A431 and PC3 cells, both obtained from the American Type Culture Collection (ATCC).

The anti-EGFR affibody molecule containing C-terminal cysteine was produced as described earlier [23]. Maleimido-DFO (Figure 1), was site-specifically conjugated to C-terminal cysteine of ZEGFR:2377 to provide DFO-ZEGFR:2377 according to the method described by Garousi and co-workers [31]. The identity of conjugates was confirmed by mass spectrometry as described earlier [31]. The purity of the conjugate was greater than 95%, as determined using reversed phase HPLC according to a method described earlier [31].

Statistical treatment and linear regression analysis were performed using GraphPad Prism software version 5.00 for Windows (GraphPad Software, San Diego, CA, USA). A two-tailed unpaired *t*-test was used for comparison of the two sets of data. The difference was considered significant when the *p* value was less than 0.05.

### 2.1. Production of ^66^Ga

Gallium-66 (^66^Ga) was produced using the ^66^Zn(p,n)^66^Ga reaction by irradiating isotopically enriched (99.07%) ^66^Zn target with 14.3 MeV protons, as described earlier [41] with 1 M [^66^Zn]Zn(NO_3_)_2_ in 0.3 M HNO_3_ as a target material. Irradiations (70–75 min, ~25 µA) were performed with a GE PETtrace cyclotron (GE Healthcare, Uppsala, Sweden) using a liquid target developed for a cyclotron production of ^68^Ga. Separation of [^66^Ga]GaCl_3_ was performed using the GE FASTlab Developer platform using methodology developed for ^68^Ga purification [41]. The product was collected here in several 300 μL fractions and the eluate with the highest radioactivity concentration was used for labelling.

### 2.2. Radiolabelling and In Vitro Stability

For labelling with ^66^Ga, the DFO-ZEGFR:2377 was reconstituted in water to obtain a concentration of 2 mg/mL. The DFO-ZEGFR:2377 solution (30 µg, 15 µL) was mixed with 80 μL of 1.25 M of NaOAc, pH 3.6. The radionuclide solution (50–100 μL, 15 MBq) was added; the mixture was thoroughly vortexed and incubated for 10 min at 85 °C. Radiochemical yield and purity of the labelled conjugate were analyzed using ITLC-SG (Agilent Technologies) developed with PBS (affibody: Rf = 0.0; other forms of ^66^Ga: Rf = 1.0). To further cross-validate radio-ITLC data, reverse phase-HPLC conducted on an Elite LaChrom system (Hitachi, VWR, Darmstadt, Germany) consisting of an L-2130 pump, a UV detector (L-2400), and a radiation flow detector (Bioscan, Washington, DC, USA) coupled in series was used. Purity analysis of labelled compounds was performed using an analytical column (Phenomenex, Aschaffenburg, Germany; Luna® 5 µm C18, 100 Å; 150 × 4.6 mm column). HPLC conditions were as follows: A = 10 mM TFA/H_2_O; B = 10 mM TFA/acetonitrile; UV-detection at 220 nm; gradient elution: 0–15 min at 5 to 70% B, 15–18 min at 70 to 95% B, 19–20 min at 5% B; and flow rate of 1.0 mL/min. The retention time of [^66^Ga]Ga-DFO-ZEGFR:2377 is 11.2 min.

Labelling of DFO-ZEGFR:2377 with ^68^Ga was performed using the method described earlier [28]. A generator eluate (120 μL, 100 MBq) was used. DFO-ZEGFR:2377 was labelled with ^89^Zr at 85^°^C according to the protocol described earlier [32].

In Vitro stability of [^66^Ga]Ga-DFO-ZEGFR:2377 was evaluated in PBS and also in the presence of 1000-fold molar excess of ethylenediaminetetraacetic acid (EDTA). Namely, after purification, samples of freshly labelled conjugate (1.4 µg, 50 µL) were mixed with EDTA (60 µg, 2 mg/mL in PBS) to obtain a 1000-fold molar excess of EDTA and incubated at room temperature for 1 h. Control samples were mixed with an equal volume of PBS. The experiment was performed in triplicate.

### 2.3. In Vitro Studies

For cell studies, two EGFR-expressing cell lines with different levels of EGFR expression, epidermoid carcinoma A431 (1.2 × 10^6^ receptors/cell) [42] and prostate carcinoma PC3 (10^5^ receptors/cell) [43] (ATCC) were used. The cell lines were cultured in McCoy’s medium, supplemented with 10% fetal bovine serum (Sigma-Aldrich, St. Louis, MI, USA), 1% L-glutamine, and PEST (penicillin 100 µ/mL and 100 µg/mL streptomycin), from Bookroom AG (Berlin, Germany). The cells were cultured at 37 °C in a humidified incubator with 5% CO_2_.

For a binding specificity test, a set of nine dishes containing approximately 10^6^ cells/dish was used for each data point. To saturate the EGFR, a 100-fold molar excess of anti-EGFR antibody cetuximab or non-labelled DFO-ZEGFR:2377 were used as blocking agents in three control dishes. Cells were incubated with blocking agents at room temperature for 15 min. Then, [^66^Ga]Ga-DFO-ZEGFR:2377 (5 nM) was added to all dishes followed by incubation for 1 h at 37 °C. After incubation, the medium was aspired. Next, the cells were washed with cold serum-free medium, treated with 2 mL trypsin–EDTA solution per dish at 37 °C, and collected. Radioactivity of cells was measured. The experiments were performed in triplicate.

To evaluate the binding affinity of [^66^Ga]Ga-DFO-ZEGFR:2377 to EGFR receptors, kinetics of labelled conjugate binding to- and their dissociation from A431 cells were measured using a LigandTracer Yellow instrument (Ridgeview Instruments AB, Vänge, Sweden). A431 cells were seeded on a local area of a cell culture dish (NunclonTM, Size 100620, NUNC A/S, Roskilde, Denmark). The measurements were performed at room temperature to prevent internalization. Uptake curves were recorded at 0.33, 1, and 3 nM of [^66^Ga]Ga-DFO-ZEGFR:2377 conjugate. Thereafter, the radioactive medium was withdrawn, fresh non-radioactive medium was added, and the dissociation curve was recorded. The data were analyzed using the InteractionMap software (Ridgeview Diagnostics AB, Uppsala, Sweden) in order to calculate the association rate, dissociation rate, and dissociation constant at equilibrium (K_D_). Analysis was performed in duplicates.

Cellular processing of bound [^66^Ga]Ga-DFO-ZEGFR:2377 was evaluated using A431 and PC3 cell lines. The cells (10^6^ cells/dish) were incubated with 5 nM of [^66^Ga]Ga-DFO-ZEGFR:2377 at 37 °C. After 1, 2, 4, 8, and 24 h incubation, the internalized fraction was determined by the acid wash method [44]. The membrane-bound conjugate was removed from cells by treatment with 4 M urea solution in a 0.1 M glycine buffer, pH 2.5, for 5 min on ice. The cell debris containing the internalized conjugates was detached by treatment with 1 M NaOH. Radioactivity of the samples was measured and the percentage of membrane-bound, internalized, and total radioactivity was calculated. The experiments were performed in triplicate.

### 2.4. In Vivo Studies

Animal studies were planned and performed in agreement with EU Directive 2010/63/EU for animal experiments and Swedish national legislation concerning protection of laboratory animals. Experiments were approved by the Ethics Committee for Animal Research in Uppsala (C4/16). Biodistribution studies were performed in female BALB/C nu/nu mice purchased from Scanbur A/S (Karlslunde, Denmark).

EGFR-expressing xenografts were established by subcutaneous injection of 10^7^ A431 cells in the hind legs of mice. The tumors were grown for 10 days. At the time of the experiment, the average animal weight was 18 ± 1 g and the average tumor weight was 0.5 ± 0.2 g. The animals were randomized into groups of four mice for each data point.

The biodistribution of [^66^Ga]Ga-DFO-ZEGFR:2377 was measured at 3, 6, and 24 h post injection (p.i.). Namely, two groups of mice were intravenously (tail vein) injected with [^66^Ga]Ga-DFO-ZEGFR:2377 (38 µg, 100 kBq/mouse) in 100 µL PBS to measure biodistribution at 3 and 6 h p.i. whereas one group was injected with 150 kBq/mouse and 38 µg, in 100 µL PBS to measure the biodistribution at 24 h p.i. In vivo specificity of the conjugate was investigated using saturation of EGF receptors in a group of mice by subcutaneous injection of 10 mg of cetuximab 24 h before injection of [^66^Ga]Ga-DFO-ZEGFR:2377 (38 µg/100 kBq in 100 µL PBS) and the biodistribution was measured 3 h p.i. For comparison, a group of mice was intravenously injected with [^68^Ga]Ga-DFO-ZEGFR:2377 (38 µg, 800 kBq/mouse) in 100 µL PBS and the biodistribution was measured 3 h p.i. Another group of mice was intravenously injected with [^89^Zr]Zr-DFO-ZEGFR:2377 (38 µg, 40 kBq/mouse) in 100 µL PBS, and the biodistribution was measured at 24 h.

The injected activity was selected based on results of previous studies in order to provide a counting statistics error of less than 3% for samples with the lowest uptake, and a dead time of less than 20% for the standards of injected activity. The injected protein dose was determined in earlier studies to be optimal for a partial saturation of EGFR in the liver without saturation of receptors in the tumor [23]. To obtain the desirable protein dose, non-labelled DFO-ZEGFR:2377 was added to conjugates during formulation.

For biodistribution measurements, the mice were euthanized by an intraperitoneal injection of an anesthetic solution (20 μL of solution per gram of body weight: ketamine, 10 mg/mL; Xylazine, 1 mg/mL) followed by heart puncture. Blood, salivary glands, lung, liver, spleen, colon, kidneys, tumor, muscle, and bone were collected and weighed. Then, the organ radioactivity was measured and uptake values of organs were calculated as a percentage of injected dose per gram tissue (%ID/g).

Small animal PET imaging was performed to obtain qualitative visual confirmation of the results of ex vivo measurements. Three mice with A431 xenografts were injected with 1 MBq of [^66^Ga]Ga-DFO-ZEGFR:2377 (38 µg) intravenously. Whole body PET and MRI measurements were performed using a 3 Tesla nanoScan PET/MRI system (Mediso Medical Imaging Systems Ltd., Budapest, Hungary) at 3, 6, and 24 h p.i. The net measurement time was one hour for the PET scans started 3 and 6 h after injection or 1.5 h for the scans performed 24 h p.i. Measured data were reconstructed using the Tera-Tomo™ 3D reconstruction engine which takes into account the matching MR images for correction of positron range. Mice were kept under general anesthesia (0.06% sevoflurane; 50%/50% medical oxygen: air) during PET/MRI acquisitions made 3 and 6 h p.i. Mice were euthanized prior to the scans 24 hours p.i. MR images were measured using a T1-weighted gradient echo sequence prior each PET scan. Acquisition time was 4 min 2 sec for 21 slices. Parameters were as follows: coronal slices (1 mm thickness), FOV 80 × 60 mm, acquisition matrix 256 × 192, resolution in plane 0.313 × 0.313 mm, 4 accumulations, repetition time 300 ms, echo time 4.5 ms, flip angle 45^o^, receiver bandwidth 40 000 Hz. Additionally, one mouse was pre-injected with 10 mg cetuximab 24 h before injection of 1 MBq of [^66^Ga]Ga-DFO-ZEGFR:2377, and imaging was performed 3 h p.i.

## 3. Results

### 3.1. Nuclide Production and Radiolabelling

^66^Ga was produced with end-of-bombardment (EOB) yields up to 0.50 GBq, which corresponds to saturation yields of 0.25 GBq/μA. The isolated [^66^Ga]GaCl_3_ activity considering all fractions was ~320–340 MBq, of which the fractions used for radiolabeling had activity concentrations ranging within 160–280 MBq/mL

The ^66^Ga labelling of ZEGFR:2377 containing DFO as a chelator was performed at 85 °C. The radiochemical yield was 64.5 ± 3.5% and the isolated yield was 61.5 ± 0.7%. The radiochemical purity of the radiolabeled affibody molecules after purification using NAP-5 columns was greater than 99%. The specific activity of [^66^Ga]Ga-DFO-ZEGFR:2377 was 0.3 MBq/µg (molar activity 2.43 GBq/µmol).

After the purification, in vitro stability test of DFO-ZEGFR:2377 labelled with ^66^Ga was performed in PBS and in the presence of 1000-fold molar excess of EDTA. The release of the ^66^Ga when challenged by EDTA was less than 4% compared to control samples incubated in PBS.

Labelling of DFO-ZEGFR:2377 with ^68^Ga and ^89^Zr was performed according to previously published protocols [28,32] and provided radiochemical purity over 98%.

### 3.2. In Vitro Studies

**Figure 2:**
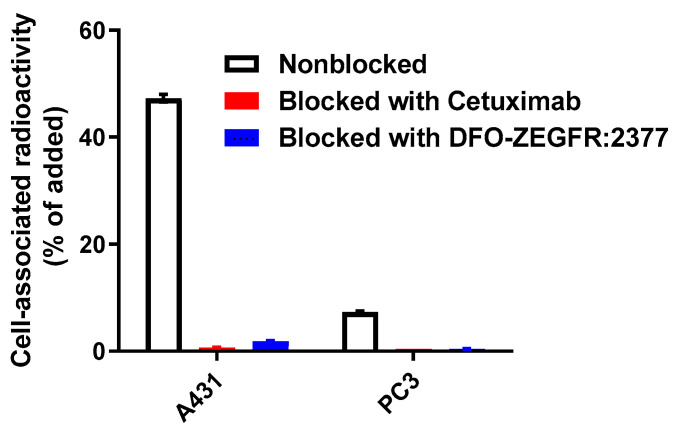
In Vitro specificity of [^66^Ga]Ga-DFO-ZEGFR:2377 conjugate bound to A431 and PC3. Cells were incubated with 5 nM [^66^Ga]Ga-DFO-ZEGFR:2377. A total of 500 nM of blocking agent was used to confirm the epidermal growth factor receptor (EGFR)-specificity of the conjugate. The data are presented as the average (*n* = 3) and SD.

The specificity of [^66^Ga]Ga-DFO-ZEGFR:2377 binding to EGFR expressing cells was tested by pre-saturation of receptors. Saturation of the receptors with a large excess of anti-EGFR antibody cetuximab and non-labelled DFO-ZEGFR:2377 affibody molecule significantly (*p* < 5 × 10^−7^) decreased the binding of the radiolabelled affibody molecules to EGFR-expressing cell lines A431 as well as PC3 cells (Figure 2). Therefore, the specificity test demonstrated that the binding of the tracer to both EGFR-expressing cell lines was EGFR-mediated. In addition, cell-associated activity was proportional to the EGFR expression level (Figure 2).

The data concerning the kinetic evaluation of binding and affinity of [^66^Ga]Ga-DFO-ZEGFR:2377 to A431 cells are presented in Table 1 and Figure S1. According to LigandTracer measurements, the best fit of the binding to A431 cell line was achieved using a 1:2 model. Interaction Map calculations showed a major strong interaction with K_D1_ = 342 ± 14 pM and a weaker minor interaction with K_D_ in the nanomolar range (K_D2_ = 29.2 ± 0.2 nM).

**Figure 3:**
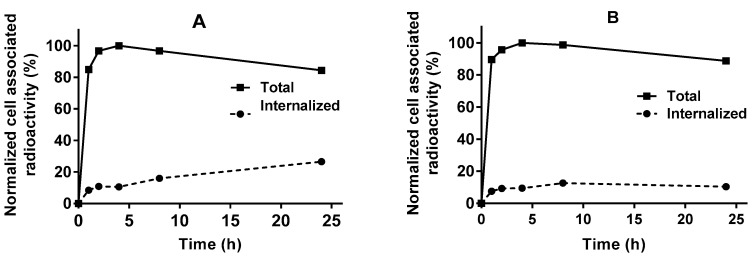
Cellular processing of [^66^Ga]Ga-DFO-ZEGFR:2377 by (**A**) A431, and (**B**) PC3 cells. Cells were incubated with 5 nM of [^66^Ga]Ga-DFO-ZEGFR:2377 conjugate. Cell-associated activity was normalized to the maximum uptake. The data are presented as an average (*n* = 3) and SD. Error bars might not be visible as they are smaller than the symbols.

The processing of bound [^66^Ga]Ga-DFO-ZEGFR:2377 by A431 and PC3 cell lines was studied by the acid wash method. The data are presented in Figure 3, whereby the labelled conjugate showed a rapid binding in the first 2 h with relatively slow internalization as it is typical for affibody molecules. The internalized fraction was less than 22% for both cell lines.

### 3.3. In Vivo Studies

**Figure 4:**
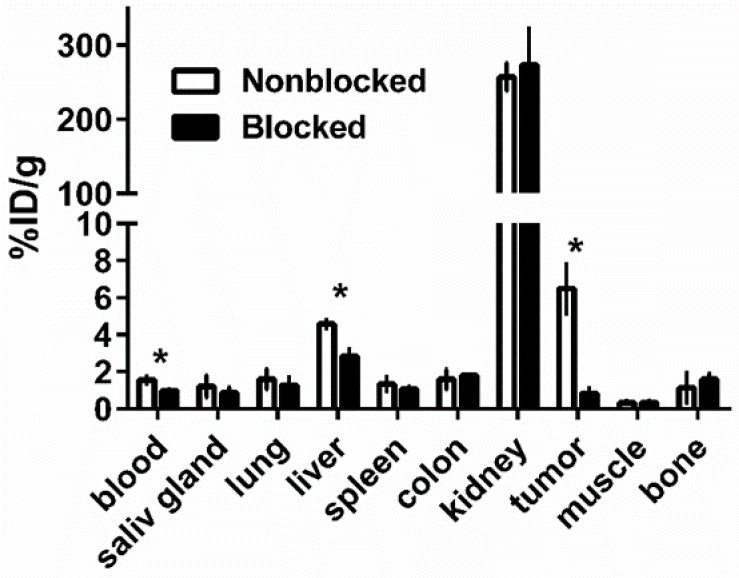
In vivo specificity of [^66^Ga]Ga-DFO-ZEGFR:2377 in A431 xenografts and EGFR-expressing organs in mice at 3 h after injection. In the blocked group, receptors were saturated by pre-injection of 10 mg of anti-EGFR antibody cetuximab 24 h before injection of labelled conjugate. The data are presented as the average (*n* = 4) and SD. Asterisk (*) marks significant (*p* < 0.005) difference.

The in vivo specificity of [^66^Ga]Ga-DFO-ZEGFR:2377 binding to EGFR in tumor xenografts was evaluated by pre-saturation of receptors using the monoclonal anti-EGFR antibody cetuximab, which binds to the same epitope as ZEGFR:2377 (Figure 4). Tumor uptake in the blocked group was significantly (*p* < 0.005) reduced compared with the unblocked group, indicating highly EGFR specific accumulation of [^66^Ga]Ga-DFO-ZEGFR:2377 in EGFR-expressing tumors. In addition, the liver uptake showed significant reduction (*p* < 0.005) in the blocked group compared with the unblocked group.

The results of the biodistribution of [^66^Ga]Ga-DFO-ZEGFR:2377 at 3, 6, and 24 h after injection in A431 xenograft-bearing mice are presented in Table 2. The clearance of radioactivity from blood and non-target tissues over time was quite fast. For example, the blood concentration was notably less than 2% at 3 h after injection, and there was a significant (*p* < 0.05) reduction of radioactivity accumulation in blood, liver, spleen, and kidneys between 3 and 24 h after injection (Table 2). A remarkable feature was the relatively rapid kidney clearance. There was no significant difference in tumor uptake at 3 h (6.5 ± 1.3% ID/g) compared to 6 h (5.1 ± 0.5% ID/g) after injection, but there was a significant (*p* < 0.05) reduction of tumor uptake at 24 h (3.7 ± 0.5% ID/g) compared with 3 and 6 h p.i. The trend of slower reduction of tumor uptake accompanied by faster clearance from blood, liver, and kidneys over time resulted in significantly (*p* < 0.05) higher tumor-to-blood, tumor-to-liver, and tumor-to-kidney ratios at 24 h compared with 3 and 6 h (Table 2).

Table 3 and Table 4 showed the data regarding the biodistribution and tumor-to-organ ratio of [^68^Ga]Ga-DFO-ZEGFR:2377 and [^89^Zr]Zr-DFO-ZEGFR:2377 in A431 xenograft-bearing mice at 3 and 24 h after injection, respectively.

**Figure 5:**
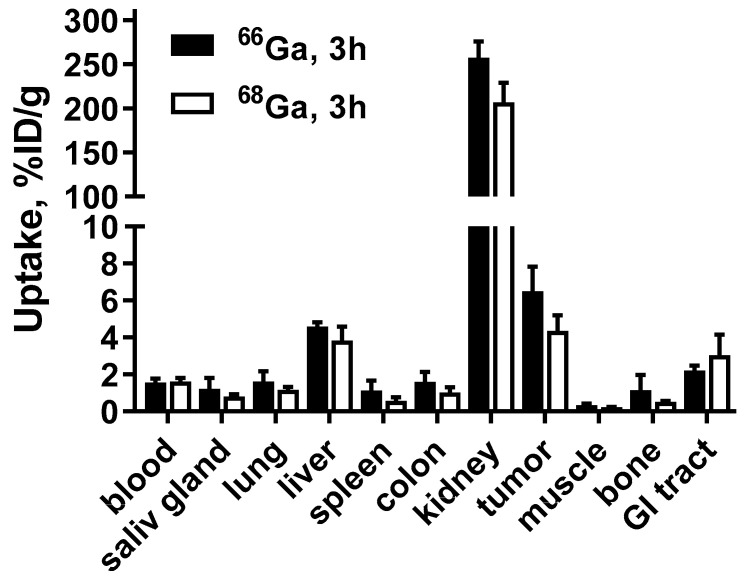
Biodistribution of [^66^Ga]Ga-DFO-ZEGFR:2377 and [^68^Ga]Ga-DFO-ZEGFR:2377 conjugates in BALB/C nu/nu mice bearing EGFR-expressing A431 xenografts at 3 h p.i.. The data are presented as the average (*n* = 4) and SD.

Figure 5 compares the biodistribution of [^66^Ga]Ga-DFO-ZEGFR:2377 and [^68^Ga]Ga-DFO-ZEGFR:2377 in A431 expressing xenografts at 3 h p.i.. As anticipated, the biodistribution of both variants was similar.

**Figure 6:**
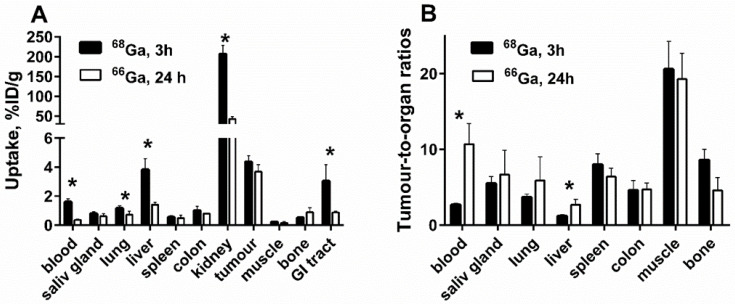
Comparison of biodistribution (**A**) and tumor-to-organ ratio (**B**) of [^68^Ga]Ga-DFO-ZEGFR:2377 and [^66^Ga]Ga-DFO-ZEGFR:2377 BALB/C nu/nu mice bearing EGFR-expressing A431 xenografts at 3 and 24 h p.i., respectively. The data are presented as the average (*n* = 4) and SD. Asterisk (*) marks significant (*p* < 0.05) difference.

A comparison of the biodistribution and tumor-to-organ ratio of [^66^Ga]Ga-DFO-ZEGFR:2377 at 24 h p.i. and [^68^Ga]Ga-DFO-ZEGFR:2377 at 3 h p.i. is shown in Figure 6. Evidently, an extension of clearance time from 3 to 24 h resulted in significantly lower uptake of [^66^Ga]Ga-DFO-ZEGFR:2377 in the blood, liver and kidney compared with [^68^Ga]Ga-DFO-ZEGFR:2377 at the earlier time point. This resulted in significantly (*p* < 0.05) higher tumor-to-blood (10.7 ± 2.7 vs. 2.7 ± 0.2), tumor-to-liver (2.7 ± 0.7 vs. 1.2 ± 0.2) and tumor-to-kidney (0.088 ± 0.014 vs. 0.021 ± 0.001) ratios provided by [^66^Ga]Ga-DFO-ZEGFR:2377 at 24 h compared with values for [^66^Ga]Ga-DFO-ZEGFR:2377 at 3 h after injection.

**Figure 7:**
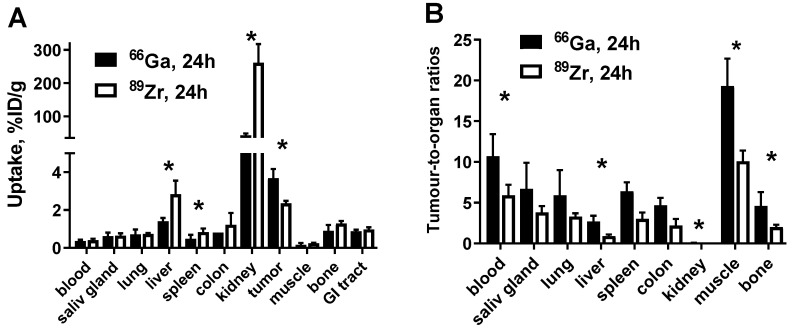
Biodistribution (**A**) and tumor-to-organ ratio (**B**) of [^66^Ga]Ga-DFO-ZEGFR:2377 and [^89^Zr]Zr-DFO-ZEGFR:2377 conjugates in BALB/C nu/nu mice bearing EGFR-expressing A431 xenografts at 24 h after injection. The data are presented as the average (*n* = 4) and SD. Asterisk (*) marks significant (*p* < 0.05) difference.

A direct comparison between [^66^Ga]Ga-DFO-ZEGFR:2377 and [^89^Zr]Zr-DFO-ZEGFR:2377 at 24 h p.i. is shown in Figure 7. [^66^Ga]Ga-DFO-ZEGFR:2377 had significantly (*p* < 0.05) higher uptake in tumors, but significantly lower uptake in the liver, spleen, and kidneys (Figure 7A). In particular, the ^66^Ga-labelled counterpart demonstrated significantly (*p* < 0.05) higher tumor-to-blood (10.7 ± 2.7 vs. 5.9 ± 1.3), tumor-to-liver (2.7 ± 0.7 vs. 0.9 ± 0.2), tumor-to-kidney (0.088 ± 0.014 vs. 0.009 ± 0.002), tumor-to-muscle (19 ± 3 vs. 10 ± 1) and tumor-to-bone (4.6 ± 1.7 vs. 2.0 ± 0.3) ratios than [^89^Zr]Zr-DFO-ZEGFR:2377 (Figure 7B).

**Figure 8:**
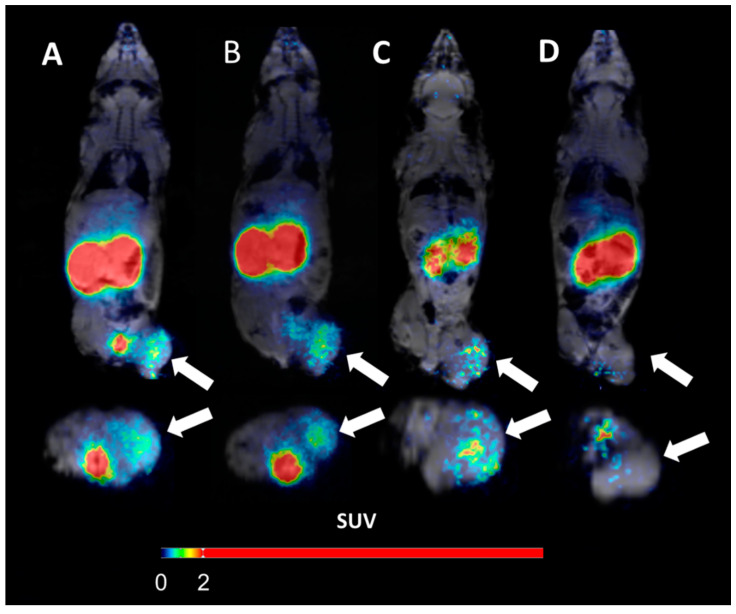
microPET/MRI imaging of EGFR-expression in A431 xenografts using [^66^Ga]Ga-DFO-ZEGFR:2377 at 3 h (**A**), 6 h (**B**), and 24 h (**C**) p.i. To confirm the in vivo specificity of [^66^Ga]Ga-DFO-ZEGFR:2377, EGF receptors were saturated in one animal (Panel (**D**)) by subcutaneous injection of 550 mg/kg cetuximab 24 h before injection of [^66^Ga]Ga-DFO-ZEGFR:2377 and imaging was performed at 3 h after tracer injection. Arrows point at tumors.

The ability to use [^66^Ga]Ga-DFO-ZEGFR:2377 for specifically visualizing EGFR-expressing xenografts was confirmed using microPET/MRI (Figure 8A–C). Mice bearing A431 xenografts were imaged 3, 6, and 24 h after injection of [^66^Ga]Ga-DFO-ZEGFR:2377. EGFR-expressing xenografts on the right hind leg were clearly visualized after injection at all three time points. The imaging results were in good agreement with the biodistribution data. There was a tendency towards reduced liver uptake with time, thus resulting in a higher tumor-to-liver ratio at the latest time point. The only normal organ with a high radioactivity accumulation was the kidneys. Otherwise, the uptake of radioactivity in the tumor appreciably exceeded uptake when compared with other normal organs, thus providing a clear, high-contrast image of an EGFR-expressing xenograft. In the control mouse, EGF receptors were saturated by pre-injection of a large molar excess of the anti-EGFR antibody cetuximab. The activity uptake in tumor was noticeably reduced, which confirmed EGFR-specificity of [^66^Ga]Ga-DFO-ZEGFR:2377 using in vivo imaging (Figure 8, panel D).

## 4. Discussion

The use of small scaffold proteins, such as affibody molecules, offers an advantage in radionuclide molecular imaging compared with the use of monoclonal antibodies due to the potential for affibody molecules to provide higher contrast [45]. Consequently, the sensitivity of such imaging is also higher. Typically, high-contrast imaging is achieved on the same day as the injection [21,45], and short-lived positron emitters (e.g., ^18^F and ^68^Ga) are generally suitable for affibody molecule labelling. Being positron emitters, the use of such radionuclides additionally enable higher sensitivity and resolution imaging by PET vs. SPECT. However, imaging of EGFR is somewhat different. Expression of EGFR in normal tissues (most importantly in the liver) and reversible binding of affibody molecules to EGFR causes slower clearance and necessitates next-day imaging in order to obtain adequate contrast. Thus, the half-life of a nuclide for labelling should be sufficiently long to prevent decay prior to a tracer’s optimal imaging time.

The use of a positron-emitting radionuclide remains to be desirable due to advantages of PET as a radionuclide imaging modality. ^66^Ga is one of a few positron emitting nuclides that meet the requirement of a sufficiently long half-life, reasonably abundant positron decay branching ratio and feasibility of production by low-energy cyclotrons available to the PET community [46] (Table S1). In addition to the selection of a radionuclide with a suitable half-life, the selection of suitable labelling chemistry is an essential factor for development of an imaging probe. Multiple studies have demonstrated that a combination of radionuclide and chelator can profoundly influence the biodistribution of affibody molecules and therefore the imaging contrast [22,47]. For example, the use of the versatile and commonly used chelator DOTA for labelling of ZEGFR:2377 with ^68^Ga resulted in higher uptake in the liver vs. tumor, thus making imaging of frequently encountered hepatic metastases impossible [27]. On the other hand, the use of DFO as a chelator provided a targeting conjugate with higher uptake in tumor than in liver [28]. Therefore, DFO was selected for labelling with ^66^Ga in this study.

This study demonstrated that DFO-ZEGFR:2377 can be labelled with ^66^Ga providing radiochemical purity sufficient for clinical application. A production of sufficient amount of radionuclide is essential for future clinical translation. In the case of ^68^Ga-labelled PSMA-binders and somatostatin analogues, an injected activity of 100–120 MBq is adequate for clinical applications. Our current experimental protocol for production of ^66^Ga enables production of about 300 MBq with a radioactivity concentration suitable for labelling. With an isolated yield of 61.5 ± 0.7%, [^66^Ga]Ga-DFO-ZEGFR:2377 with activity of 160–180 MBq could be produced after single irradiation. In terms of positron emission such activity would be equivalent to 100–115 MBq of ^68^Ga. Furthermore, the production rate of ^66^Ga can be appreciable increased by rising the proton beam current form 25 to 40 µA on the existing liquid technology, or by use of solid targets. Taking into account that a clinically relevant injected mass of DFO-ZEGFR:2377 should be on the order of several milligrams to prevent sequestering of the imaging probe by the liver, we consider the current specific activity as sufficient for clinical translation. [^66^Ga]Ga-DFO-ZEGFR:2377 retained specific binding to EGFR-expressing cell lines since this binding was saturable with both non-labelled ZEGFR:2377 or anti-EGFR antibody cetuximab (Figure 2). According to InteractionMap analysis, the binding of [^68^Ga]Ga-DFO-ZEGFR:2377 to A431 cells was characterized by high affinity (K_D1_ = 342 ± 15 pM) interaction, which was predominant (Table 1). There was also a weaker (K_D1_ = 29.2 ± 0.2 nM) but much less abundant interaction. It has been previously shown that the appearance of a weaker interaction is associated with the presence of dimerized EGFR on cancer cells’ surface [48]. The internalization of [^66^Ga]Ga-DFO-ZEGFR:2377 conjugate by cancer cells after binding was slow (Figure 3), similarly to the pattern observed for [^68^Ga]Ga-DFO-ZEGFR:2377 [28].

Saturation of EGFR in A431 xenografts resulted in significant (*p* < 0.05) reduction in [^66^Ga]Ga-DFO-ZEGFR:2377 uptake in tumors (Figure 4 and Figure 8, Panels A and D). This demonstrated that EGFR-specific targeting was preserved in vivo.

The biodistribution data (Table 2) demonstrated that uptake of [^66^Ga]Ga-DFO-ZEGFR:2377 at 3 h p.i. was already higher than in the majority of other organs. However, the renal uptake was higher than the tumor uptake, which is attributed to an efficient re-absorption in proximal tubuli. The high renal uptake is typical for affibody molecules [22]. However, clinical studies with HER2-targeting affibody molecules [49] demonstrated that the high renal uptake does not complicate imaging of metastases in the lumbar area, including adrenal metastases. An interesting finding was a rapid clearance of activity from kidneys (i.e., six-fold between 3 and 24 h). This is unusual for radiometal-labelled affibody molecules. In the case of [^111^In]In-DOTA-ZEGFR:2377, [23] the renal activity reduced only 1.3-fold between 4 and 24 h p.i.; in the case of [^57^Co]Co-DOTA-ZEGFR:2377 [27], the reduction between 3 and 24 h was 1.2-fold. The renal retention of activity was much lower for [^66^Ga]Ga-DFO-ZEGFR:2377 in this study (Table 2 and Figure 8A–C). Such a rapid clearance suggests that the residualizing properties of the [^66^Ga]Ga-DFO-label are not very strong. Accordingly, there was also some release of [^66^Ga]Ga-DFO-ZEGFR:2377 activity from tumors (6.5 ± 1.3% ID/g at 3 h p.i. and 3.7 ± 0.5% ID/g at 24 h p.i.). However, the retention of activity in tumor was sufficient to provide a significant (*p* < 0.05) increase in tumor-to-blood and tumor-to-liver and tumor-to-kidney ratios at 24 h compared with 3 h p.i. (Table 2). This is essential as blood-borne activity contributes to background in all tissues, and the liver often harbors metastases. Thus, the use of a long-lived radionuclide permits improvement in the imaging contrast and therefore, the sensitivity of the imaging of EGFR.

Expression of EGFR in the liver of young rodents can differ depending on their age [50]. These changes are difficult to control. Accordingly, an injected protein dose that was optimal in one study might result in an excessive breakthrough of a radioligand through the “liver barrier” and partial saturation of receptors in tumor leading to decreased tumor uptake in another study. Particularly, the tumor uptake of [^68^Ga]Ga-DFO-ZEGFR:2377 in this study was nearly two-fold lower than in the previous one [28]. This resulted in a notable reduction of tumor-to-blood and tumor-to-muscle ratios compared with previous data. However, the comparison between [^66^Ga]Ga-DFO-ZEGFR:2377, [^68^Ga]Ga-DFO-ZEGFR:2377 and [^89^Zr]Zr-DFO-ZEGFR:2377 in this study was performed head-to-head, in the same batch of tumor-bearing mice. This permits a reliable comparison between tested compounds since the batch-to-batch variability of the animal physiology or expression of EGFR in normal organs influences all imaging probes in a similar fashion. For [^68^Ga]Ga-DFO-ZEGFR:2377, a 3 h p.i. time point was selected for comparison. This is nearly three half-lives of the radionuclide. Performing imaging any later would necessitate injection of higher activity, which would increase an absorbed dose burden to patients. Data from this study showed (Figure 7B) that imaging at 24 h using [^66^Ga]Ga-DFO-ZEGFR:2377 would result in appreciably higher tumor-to-blood, tumor-to-liver and tumor-to-bone ratios compared with [^89^Zr]Zr-DFO-ZEGFR:2377 at the same time point.

^89^Zr has a sufficiently long half-life (78.4 h) to permit imaging at 24 h p.i. This radionuclide is commercially available, which simplifies its application for imaging. DFO is a commonly used chelator for labelling of proteins with ^89^Zr [51]. We have demonstrated that the use of [^89^Zr]Zr-DFO-ZEGFR:2377 permits higher contrast in imaging of EGFR expression in xenografts than ^89^Zr-labelled antibody cetuximab [31]. However, our earlier studies [28] have demonstrated that 3 h p.i., [^68^Ga]Ga-DFO-ZEGFR:2377 provides higher tumor-to-organ ratio. This study has demonstrated that [^66^Ga]Ga-DFO-ZEGFR:2377 provides significantly (*p* < 0.05) higher tumor-to-blood, tumor-to-liver, tumor-to-kidneys, tumor-to-muscle, and tumor-to-bone ratios than [^89^Zr]Zr-DFO-ZEGFR:2377 at 24 h p.i. (Figure 7B). Thus, ^66^Ga is a more suitable radionuclide for imaging using DFO-ZEGFR:2377 at 24 h after injection than ^89^Zr.

## 5. Conclusions

The use of the intermediate half-life positron emitter ^66^Ga for labelling of DFO-ZEGFR:2377 permits PET imaging of EGFR expression at 24 h after injection. The results of this comparative evaluation demonstrated that the use of ^66^Ga could improve PET imaging contrast compared with the use of short-lived ^68^Ga by imaging at later time point, which permits activity clearance from non-specific compartments. At 24 h after injection, [^66^Ga]Ga-DFO-ZEGFR:2377 provides better contrast compared with [^89^Zr]Zr-DFO-ZEGFR:2377. Thus, selection of a radionuclide with an optimal half-life and chemical properties can appreciably improve imaging properties of EGFR-targeting affibody molecules.

## Figures and Tables

**Table 1:** Apparent equilibrium dissociation constants (K_D_) for the interaction between [^66^Ga]Ga-DFO-ZEGFR:2377 and EGFR-expressing A431 cells determined using an Interaction Map analysis of the LigandTracer sensorgrams.

k_a1_ (1/M×s) × 10^4^	k_a1_ (1/s) × 10^−6^	K_D1_ (pM)	Weight_1_, %	k_a2_ (1/M×s) × 10^4^	k_d2_ (1/s) × 10^−3^	K_D2_ (nM)	Weight_2_, %
2.2 ± 0.1	4.0 ± 0.5	342 ± 15	74.1 ± 0.1	3.5 ± 0.1	1.03 ± 0.05	29.2 ± 0.2	8.0 ± 0.8

**Table 2:** Biodistribution and tumor-to-organ ratio for [^66^Ga]Ga-DFO-ZEGFR:2377 in BALB/C nu/nu mice bearing A431 xenografts at 3, 6, and 24 h after injection. Data are expressed as the percentage of administered activity (injected probe) per gram of tissue (% ID/g) after intravenous injection of the probe. The data are presented as the average (*n* = 4) and SD.

	Uptake, %ID/g			Tumor-to-Organ Ratio		
	3 h	6 h	24 h	3 h	6 h	24 h
Blood	1.6 ± 0.2 ^a,b^	1.2 ± 0.1 ^a,c^	0.4 ± 0.1 ^b,c^	4.2 ± 0.6 ^b^	4.2 ± 0.6 ^c^	10.7 ± 2.7 ^b,c^
Salivary gland	1.2 ± 0.6	0.7 ± 0.3	0.6 ± 0.2	5.9 ± 2.1	7.4 ± 2.7	6.7 ± 3.2
Lung	1.6 ± 0.6 ^b^	0.9 ± 0.3 ^c^	0.7 ± 0.3 ^b,c^	4.2 ± 1.0	5.1 ± 0.4	5.9 ± 3.1
Liver	4.6 ± 0.2 ^a,b^	3.5 ± 0.3 ^a,c^	1.4 ± 0.2 ^b,c^	1.4 ± 0.3 ^b^	1.5 ± 0.3 ^c^	2.7 ± 0.7 ^b,c^
Spleen	1.3 ± 0.4 ^a,b^	0.7 ± 0.2 ^a^	0.5 ± 0.2 ^b^	5.0 ± 0.3	8.2 ± 3.1	9.4 ± 5.4
Colon	1.6 ± 0.5	1.0 ± 0.3	0.811 ± 0.001	4.4 ± 1.4	5.5 ± 1.4	4.7 ± 0.9
Kidneys	257 ± 19 ^a,b^	166 ± 17 ^a,c^	42 ± 6 ^b,c^	0.025 ± 0.005 ^b^	0.031 ± 0.002 ^c^	0.088 ± 0.014 ^b,c^
Tumor	6.5 ± 1.3 ^b^	5.1 ± 0.5 ^c^	3.7 ± 0.5 ^b,c^	-	-	-
Muscle	0.3 ± 0.1	0.2 ± 0.1	0.2 ± 0.1	18 ± 1	24 ± 7	19 ± 3
Bone	1.4 ± 0.8	0.9 ± 0.3	0.9 ± 0.3	10 ± 5	6.2 ± 1.7	4.6 ± 1.7
GI *	2.2 ± 0.3	1.5 ± 0.1	0.9 ± 0.1	-	-	-
Carcass *	8.2 ± 0.9	6.2 ± 0.5	3.8 ± 0.3	-	-	-

^a^ Significant difference between 3 and 6 h; ^b^ significant difference between 3 and 24 h; ^c^ significant difference between 6 and 24 h. * Data for gastrointestinal (GI) tract with content and carcass are presented as % of injected dose per whole sample.

**Table 3:** Biodistribution and tumor-to-organ ratio for [^68^Ga]Ga-DFO-ZEGFR:2377 in BALB/C nu/nu mice bearing A431 xenografts at 3 h after injection. Data are expressed as the percentage of administered activity (injected probe) per gram of tissue (% ID/g) after intravenous injection of the probe. The data are presented as the average (*n* = 4) and SD. * Data for gastrointestinal (GI) tract with content and carcass are presented as % of injected dose per whole sample.

	Uptake, %ID/g	Tumor-to-Organ Ratio
	3 h	3 h
Blood	1.6 ± 0.2	2.7 ± 0.2
Salivary gland	0.8 ± 0.1	5.5 ± 0.9
Lung	1.2 ± 0.2	3.7 ± 0.4
Liver	3.8 ± 0.8	1.2 ± 0.2
Spleen	0.6 ± 0.1	8.0 ± 1.4
Colon	1.0 ± 0.3	4.6 ± 1.3
Kidneys	207 ± 22	0.021 ± 0.001
Tumor	4.4 ± 0.7	-
Muscle	0.22 ± 0.03	20.6 ± 3.7
Bone	0.5 ± 0.1	8.6 ± 1.4
GI *	3.0 ± 1.1	-
Carcass *	6.7 ± 1.0	-

**Table 4:** Biodistribution and tumor-to-organ ratio for [^89^Zr]Zr-DFO-ZEGFR:2377 in BALB/C nu/nu mice bearing A431 xenografts at 24 h after injection. Data are expressed as the percentage of administered activity (injected probe) per gram of tissue (% ID/g) after intravenous injection of the probe. The data were presented as the average (*n* = 4) and SD. * Data for GI tract with content and carcass are presented as % of injected dose per whole sample.

	Uptake, %ID/g	Tumor-to-Organ Ratio
	24 h	24 h
Blood	0.4 ± 0.1	5.9 ± 1.3
Salivary gland	0.6 ± 0.1	3.8 ± 0.8
Lung	0.7 ± 0.1	3.3 ± 0.4
Liver	2.8 ± 0.7	0.9 ± 0.2
Spleen	0.8 ± 0.2	3.0 ± 0.8
Colon	0.9 ± 0.1	2.2 ± 0.8
Kidneys	261 ± 56	0.009 ± 0.002
Tumor	2.4 ± 0.1	-
Muscle	0.24 ± 0.03	10.1 ± 1.3
Bone	1.3 ± 0.1	2.0 ± 0.3
GI *	1.0 ± 0.1	-
Carcass *	5.1 ± 1.3	-

## Data Availability

Data is contained within the article or supplementary material.

## Supplementary Materials

The following are available online at https://www.mdpi.com/1999-4923/13/2/292/s1, Figure S1: LigandTracer sensorgram and InteractionMap of [^66^Ga]Ga-DFO-ZEGFR:2377 binding to EGFR-expressing A431 cells. Input data were obtained from LigandTracer measurement of cell-bound activity during association of labelled conjugate to- and dissociation from A431 cells. Binding was measured at three different concentrations: 0.33, 1, and 3 nM. Measurement was performed in duplicates. Table S1: Long-lived positron-emitting radiometals. Data are taken from [51]. Emitted gamma-quanta with abundance over 5% are shown.
