# Supplementary material for: The Use of a Non-Conventional Long-Lived Gallium Radioisotope 66Ga Improves Imaging Contrast of EGFR Expression in Malignant Tumours Using DFO-ZEGFR:2377 Affibody Molecule
Source: Pharmaceutics. 2021 Feb 23;13(2):292. doi: 10.3390/pharmaceutics13020292 (PMC7926986; doi:10.3390/pharmaceutics13020292)
Supplement: Supplementary file 1 [file pharmaceutics-13-00292-s001.pdf]

pharmaceutics

Supplementary Materials: The Use of a Non-Conventional Long-Lived Gallium Radioisotope ^{66}Ga Improves Imaging Contrast of EGFR Expression in Malignant Tumours using DFO-ZEGFR:2377 Affibody Molecule

Maryam Oroujeni, Tianqi Xu, Katherine Gagnon, Sara S. Rinne, Jan Weis, Javad Garousi, Ken G. Andersson, John Löfblom, Anna Orlova, Vladimir Tolmachev

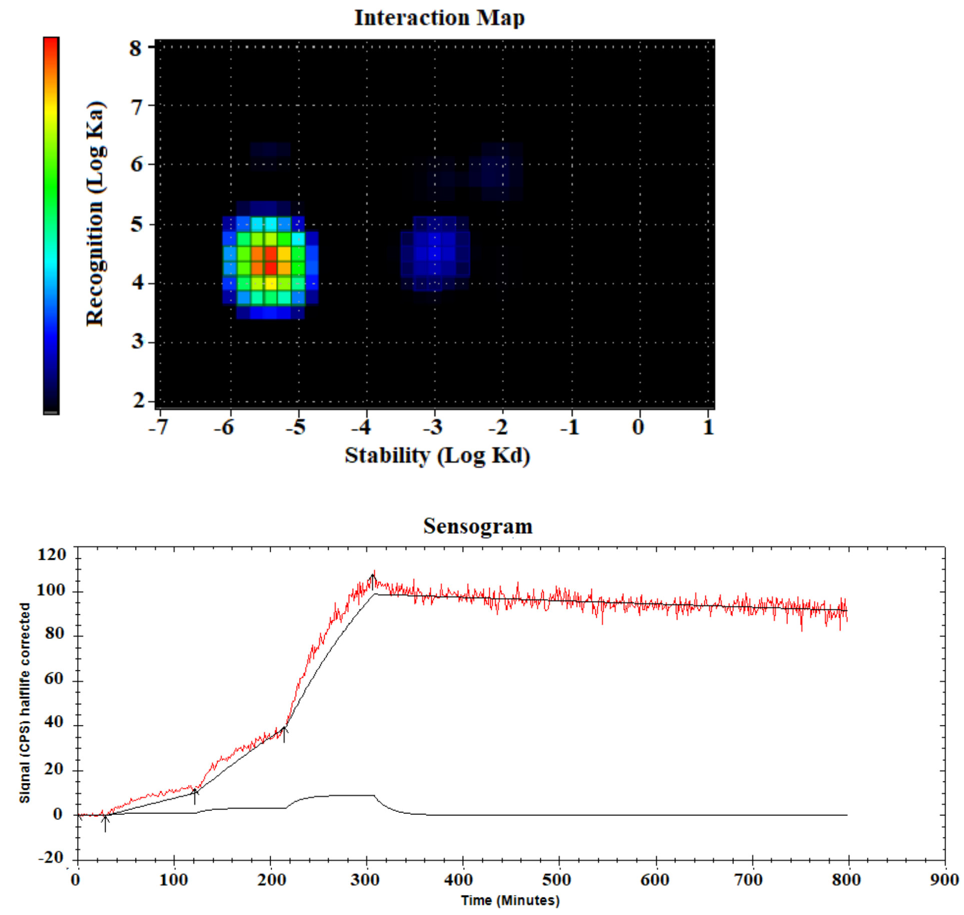

Figure S1. LigandTracer sensorgram and InteractionMap of [^{66}Ga]Ga-DFO-ZEGFR:2377 binding to EGFR-expressing A431 cells. Input data were obtained from LigandTracer measurement of cell-bound activity during association of labelled conjugate to- and dissociation from A431 cells. Binding was measured at three different concentrations: 0.33, 1, and 3 nM. Measurement was performed in duplicates.

Table S1. Long-lived positron-emitting radiometals. Data are taken from [51]. Emitted gamma-quanta with abundance over 5% are shown.

Nuclide	Half-life, hour	Mode of decay	Principal photon emissions, keV (abundance in %)
^{55}Co	17.5	β^+ 76 %<br>EC 24 %	**511(152%),** 477(20.2 %), 931(75 %), 1317(7.1%), 1408(16.9%)
^{64}Cu	12.7	β^+ 18<br>β^- 37%<br>EC 24 %	**511(36%),** 1346(0.5%),
^{66}Ga	9.49	β+ 56.5 %,<br>EC 43.5%	**511 (113%),** 834 (5.9%), 1039 (37%), 2190 (5.3%), 2751 (22.7%)
^{86}Y	14.7	β^+ 33 %<br>EC 67 %	**511(66%),** 443(16.9%), 628(32.6%), 646(9.2%), 703(15.4%), 778(22.4%), 1077(82.5%), 1153(30.5%), 1854(17.2%), 1920(20.8%)
^{89}Zr	78.4	β^+ 23%<br>EC 77%	**511(46%),** 909(100%)
